# Supplementary material for: Causal interplay between lactose intolerance and gut microbiota: a combined bidirectional Mendelian randomization and in vivo validation study
Source: Front Nutr. 2026 Jun 1;13:1803337. doi: 10.3389/fnut.2026.1803337 (PMC13265576; doi:10.3389/fnut.2026.1803337)
Supplement: Supplementary file 1 [file Data_Sheet_1.zip › supplementary materials/Forward/leave one out cross validation/ebi-a-GCST90027791.finngen_R12_E4_LACTONAS.pdf]

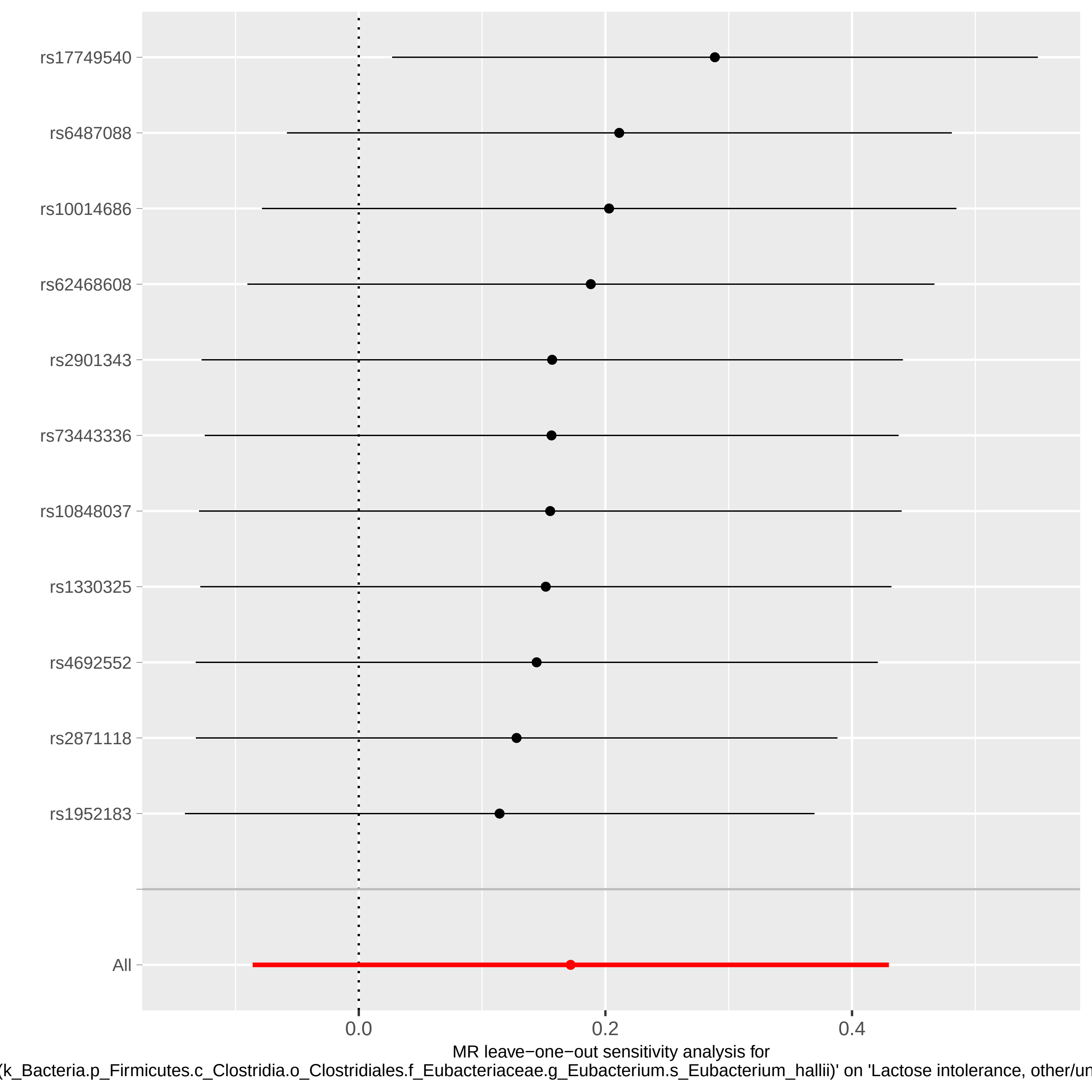

rs17749540
rs6487088
rs10014686
rs62468608
rs2901343
rs73443336
rs10848037
rs1330325
rs4692552
rs2871118
rs1952183
All
0.0
0.2
0.4
MR leave−one−out sensitivity analysis for
(k_Bacteria.p_Firmicutes.c_Clostridia.o_Clostridiales.f_Eubacteriaceae.g_Eubacterium.s_Eubacterium_hallii)' on 'Lactose intolerance, other/un
